# Current Status and Future Perspectives of Immunotherapy for Locally Advanced or Metastatic Urothelial Carcinoma: A Comprehensive Review

**DOI:** 10.3390/cancers12010192

**Published:** 2020-01-13

**Authors:** Tae Jin Kim, Kang Su Cho, Kyo Chul Koo

**Affiliations:** 1 Department of Urology, CHA University College of Medicine, CHA Bundang Medical Center, Seongnam 13496, Korea; tjkim81@cha.ac.kr; 2 Department of Urology, Gangnam Severance Hospital, Yonsei University College of Medicine, Seoul 06229, Korea; kscho99@yuhs.ac

**Keywords:** biomarkers, clinical trials, immune checkpoint inhibitor, immunotherapy, urothelial carcinoma

## Abstract

Advancements in the understanding of tumor immunology in urothelial carcinoma (UC) have led to U.S Food and Drug Administration (FDA) approval of five novel anti-programmed cell death protein-1/ligand 1 (PD-1/L1) checkpoint inhibitors. In 2017, the anti-PD-L1 antibody atezolizumab and the anti-PD-1 antibody pembrolizumab gained approval for use in cisplatin-ineligible patients with locally advanced and metastatic UC. These approvals were based on single-arm trials, IMvigor210 (atezolizumab) and KEYNOTE-052 (pembrolizumab). Since then, additional checkpoint inhibitors, including avelumab, durvalumab, and nivolumab, have gained approval. Preliminary results suggest additional benefits with combinations of these agents in both first- and subsequent-line therapies, inferring a paradigm shift in the future treatment approach in advanced UC. Ongoing clinical trials will investigate how to utilize predictive biomarkers for optimal patient selection and to incorporate immunotherapy into earlier lines of multimodal treatment. In this comprehensive review, we summarize the evidence supporting the use of checkpoint inhibitors for patients with UC, and highlight ongoing clinical trials that are investigating novel combinations of immunotherapy in various disease settings.

## 1. Introduction

Urothelial carcinoma (UC) of the urinary tract is the fourth most common type of malignancy worldwide [1]. Tobacco smoking is the most common risk factor and is responsible for 50% of all UCs. Other risk factors include pelvic radiation for other malignancies, genetic predisposition, and occupational exposure to carcinogens, including polycyclic aromatic hydrocarbons, chlorinated hydrocarbons, and aromatic amines [2].

The urinary bladder is the most common pathologic site of occurrence, followed by the renal pelvis and the ureter of the upper urinary tract and the urethra of the lower urinary tract. Despite advancements in the understanding of the underlying pathophysiology of UC, high recurrence rates in the early stages and ineffective systematic treatments for metastatic and advanced diseases are drawbacks to significant improvements in the overall prognosis of UC [3].

Recent advancements in the understanding of tumor immunology have opened new horizons for the management of UC. Knowledge of the biology of the immune system regarding checkpoint molecule inhibitors has led to the development of novel systemic agents with applications in various malignancies [4]. These agents have shown positive results in the treatment of locally advanced and metastatic UCs and are now being investigated for application in other clinical settings [4]. In this article, we perform a comprehensive review of contemporary literature to investigate evidence regarding current chemotherapy and emerging immunotherapy agents, biomarkers for predicting treatment response, and ongoing clinical trials for UC.

## 2. Classification of Urothelial Carcinoma

### 2.1. Histological Subtypes

Transitional cell carcinoma is the most common histological subtype of UC of the urinary bladder, and variant histology is identified in approximately 10% of all UCs. Accurate identification of variant histology has important implications for patient management. Unfortunately, variant histology is often under-recognized or misclassified due to evolving criteria for diagnosis and multiple synchronous variants that may exist in a single patient [5].

The 2016 World Health Organization classification provides the most updated pathological category and variant subtypes of UC based on molecular features (Table 1) [6]. Overall, treatment regimens effective for conventional UC have limited efficacy for variant histology, and patients usually exhibit an aggressive disease course. Neoadjuvant chemotherapy and subsequent radical cystectomy are recommended for patients with variant histology. Nevertheless, radical cystectomy is generally recommended as a first-line treatment due to limited data supporting the efficacy of perioperative chemotherapy or radiation therapy.

The advancements in pathological diagnosis and understanding of how different histological variants affect the disease course is changing the paradigm of UC management, especially in regard to immunotherapy [7,8]. For instance, lymphoepithelioma-like UCs are characterized as harboring prominent lymphoid stroma that includes T and B lymphocytes, histiocytes, plasma cells, and occasional neutrophils or eosinophils. Preliminary data has shown that this subset of patients exhibits higher response rates to immunotherapy than in those with conventional UCs [9,10].

### 2.2. mRNA Subtypes

The Cancer Genome Atlas (TCGA) analysis for muscle-invasive bladder cancer allowed the identification of mRNA subtypes: (1) luminal-papillary, (2) luminal-infiltrated, (3) luminal, (4) basal/squamous, and (5) neuronal [11]. The classification based on mRNA subtypes has the potential to be utilized to stratify patients for a specific treatment regimen. For instance, relatively higher mutational burden and higher antigen load were identified in the MSig1 cluster, characterized by favorable survival outcomes in this subset of patients. Improved survival observed in these patients infers the presence of a natural host immune reaction to the high antigen load that may have inhibited tumor growth and metastasis [11]. Indeed, this presumption warrants confirmation in clinical trials, preferably involving immune checkpoint inhibitors. Furthermore, the validation of this subtype as a prognosticator for the response to immunotherapy may support the use of immunotherapy in the neoadjuvant setting since a higher load of tumor antigens would be present if the primary tumor is still in-situ.

## 3. Treatment Strategy of Urothelial Carcinoma of the Urinary Bladder

UC of the urinary bladder is categorized into non-muscle invasive disease (Ta, T1, and Tis), muscle-invasive disease (≥T2), and metastatic disease, and each clinical spectrum differs in prognosis, management, and treatment strategy [3]. In general, conventional treatment options for muscle-invasive or metastatic disease included chemotherapy, radiation therapy, and radical cystectomy [2].

### 3.1. Muscle-Invasive Disease

Approximately 20% of patients with bladder carcinoma are initially diagnosed with muscle-invasive disease [12]. Muscle-invasive disease is characterized by malignant infiltration beyond the basement membrane. The treatment goal is focused on determining whether the bladder can be preserved without compromising recurrence-free survival, or whether it should be removed to maximize survival outcome.

For muscle-invasive disease without metastasis, cisplatin-based neoadjuvant chemotherapy is recommended for patients fit for radical cystectomy. For patients who have not received cisplatin-based neoadjuvant chemotherapy and have a non-organ confined disease (pT3/T4 and/or N+), adjuvant cisplatin-based chemotherapy is recommended. For patients who desire to preserve the urinary bladder, maximal debulking transurethral resection of the tumor and subsequent administration of adjuvant chemo-radiation therapy could be an option [2].

### 3.2. Metastatic Disease

Approximately 5% of patients initially present with metastatic disease, and the goal is to prolong survival without compromising the quality of life. For patients with metastatic disease, cytotoxic therapy has been the standard treatment of choice during the past half-decade [3,13]. The most common first-line chemotherapy regimens for cisplatin-eligible patients with locally advanced or metastatic disease include gemcitabine plus cisplatin (GC) and dose-dense methotrexate, vinblastine, doxorubicin, and cisplatin (M-VAC), which result in median overall survivals (OS) of 13.8 and 14.8 months, respectively [14,15]. On the other hand, a significant proportion of patients with advanced disease are cisplatin-ineligible due to negative prognostic factors such as poor Eastern Cooperative Oncology Group (ECOG) performance status, old age, impaired renal function, or significant comorbidities. Second-line chemotherapy or supportive treatment provides significantly less survival benefit, with a median OS of approximately nine months [16,17].

## 4. Chemotherapy for the Treatment of Urothelial Carcinoma

### 4.1. First-Line Chemotherapy for Cisplatin-Eligible Patients with Urothelial Cancer

Cisplatin-based chemotherapy is the initial regimen of choice for cisplatin-eligible patients with metastatic UC. The M-VAC regimen has shown a high response rate of 72% for metastatic disease and has been adopted as the standard front-line treatment [18]. Subsequent clinical trials showed that cisplatin-based combination regimens were more efficient in comparison to the administration of single-agent cisplatin [19]. However, the overall therapeutic efficacy of this combination regimen was hindered by high toxicity and low tolerability. In the early 2000s, the less toxic combination of GC was accepted as a new standard of care in the palliative setting for patients with advanced UCs.

The GC regimen failed to show a superior prolongation in OS compared to the M-VAC regimen. However, patients exhibited lower rates of neutropenia, mucositis, and resulting neutropenic fever, demonstrating that the GC regimen provided a less toxic alternative to M-VAC chemotherapy [20]. Still, only 60% of patients tolerated the first cycle. Compared to the M-VAC regimen, the GC regimen resulted in a higher prevalence of thrombocytopenia. The addition of paclitaxel to GC (PCG) is a triple combination that is administered on a three-week schedule. The PCG regimen improved response rates but failed to provide a statistically significant improvement in OS compared to the one-month schedule of the GC regimen [21].

### 4.2. First-Line Chemotherapy for Cisplatin-Ineligible Patients with Urothelial Cancer

Cisplatin-based chemotherapy is the first treatment option for locally advanced or metastatic disease. However, due to toxicity, the regimen is not well tolerated, and approximately two-thirds of the patients are ineligible [22]. The criteria for cisplatin ineligibility are ECOG performance status ≥2, grade ≥2 neuropathy or hearing loss, New York Heart Association classification III heart failure, and creatine clearance <60 mL/min. Cisplatin-ineligible patients have inferior survival outcomes compared to those receiving cisplatin. Therefore, the development of alternative therapies is imperative for this population.

For patients who are ineligible for cisplatin but who are still suitable for combination chemotherapy, carboplatin-based regimens have a crucial role. Attempts were performed to reduce the toxicity of chemotherapy by substituting carboplatin for cisplatin. The methotrexate, carboplatin, and vinblastine (MCAV) regimen were compared with the gemcitabine and carboplatin (GemCarbo) regimen for cisplatin-ineligible patients in a European Organization for Research and Treatment of Cancer trial [23]. The study population included those with poor kidney function, in addition to ECOG performance status ≥2 patients. The study showed comparable objective response rates (ORR) and OS rates between the GemCarbo and MCAV regimens (ORR 36.1% vs. 21.0%, *p* = 0.08; and OS 9.3 months vs. 8.1 months, *p* = 0.64, respectively). Of the two regimens, higher rates of severe acute toxicity such as renal toxicity, thrombocytopenia, neutropenic fever, and death were noted in the MCAV regimen compared to the GemCarbo regimen (21.2% vs. 9.3%, respectively). In patients with both poor performance and kidney function, the ORR decreased to 25% in the GemCarbo regimen and increased to 27% for the MCAV regimen, while an increase in severe toxicity rates was shown for both regimens (12.5% for the GemCarbo regimen vs. 27.3% for the MCAV regimen). The feasibility of triple combination chemotherapy has been studied in patients with renal insufficiency. A regimen consisting of gemcitabine, carboplatin, and paclitaxel was investigated in patients without a history of chemotherapy or with only one prior chemotherapy regimen. The trial enrolled patients regardless of renal function, with a cutoff value of serum creatinine of ≤2.5 mg/dL [24]. The ORR was 43%, with a median OS of 11 months. Due to the high incidences of neutropenia, this regimen was considered more toxic compared to conventional doublet-based chemotherapy.

## 5. Immunotherapy for the Treatment of Urothelial Carcinoma

### 5.1. The Rationale for Immunotherapy in Urothelial Carcinoma

The scope of immunotherapy for cancer patients has broadened tremendously with breakthroughs in the understanding of the immune system. The objective of immunotherapy is to eliminate cancer cells by augmenting the interaction between the immune and tumor cells of the host. Clinical applications of immunotherapy include boosting the immune response with exogenous cytokines, administering vaccines for tumor-associated antigens, and activating targeted antibodies on the surface proteins of immune checkpoint molecules [18]. In normal physiology, immune checkpoints suppress the adaptive immune response to prevent incorrect or prolonged T-cell activation [25]. In this process, antigen presentation to the T cells by antigen-presenting cells (APCs) is the key component. Several co-stimulatory or inhibitory proteins that permit T cells to activate the immune process have been identified. The CD28 protein plays a pivotal role in the stimulation of T cells. The binding of CD28 proteins on T cells to the proteins on APCs causes T-cell proliferation. The T cell inhibition cascade is activated after cytotoxic T-lymphocyte associated antigen 4 (CTLA-4) is bound to its ligands (B7-1 or B7-2), or when programmed death 1 (PD-1) protein binds to its PD-L1 ligand on the surface of tumor cells or APCs [25]. Cancer cells may evade the anti-tumor immune response by exploiting these immune checkpoint pathways and inhibiting the host immune cell (IC) proliferation [26].

Apart from the CTLA-4 and PD-1/PD-L1 pathways, other immune molecules, such as T cell immunoglobulin mucin-3 (TIM-3), lymphocyte activation gene-3 (*LAG-3*), and the B7 superfamily (B7-H3, B7-H4) are currently being studied as candidates for future immune checkpoints. As a regulatory molecule, TIM-3 has a crucial role in innate immune cell-mediated antitumor responses. Along with PD-L1, TIM-3 is coexpressed by CD8 tumor-infiltrating lymphocytes (TIL), which results in negative regulation of cytokine secretion [27]. The *LAG-3* molecule is located on the surface of the immune cells and plays a diverse role in T cell regulation. The protein negatively regulates the cellular proliferation and activation of T cells and has been observed to play a suppressive role in the CD4 and CD8 immune response [28]. B7-H3 and B7-H4 are part of the B7 costimulatory molecules which can be found in immune and nonlymphoid cells. The role of B7-H3 in the cancer immune-axis is controversial. Although the molecule was first characterized as a T cell activator, several studies have shown it may trigger both upregulation and downregulation of T cell function [29,30].

Evidence from various studies suggests that inhibiting the checkpoint pathway is suitable for cancers with high somatic mutation rates, which may trigger a high number of tumor-specific neo-antigens [31,32]. DNA mutations caused by cancer cells are reflected in the production burden of altered proteins, and their presence results in the priming and activation of the host immune system. In turn, they can potentially be identified as foreign antigens [33]. UC harbors the fourth highest rates of mutations of all cancers and is known to be highly antigenic [34,35]. The understanding based on these findings and principles provides the rationale for the clinical application of immunotherapy in UC.

### 5.2. First-Line Immunotherapy for Cisplatin-Ineligible Patients

Cisplatin-based chemotherapy is the treatment of choice in the first-line setting for locally advanced and metastatic UC. However, due to low tolerance and the short duration of response, an alternative approach for the treatment of UC is imperative. Two clinical trials of the anti‒PD-1/PD-L1 chemotherapy agents (atezolizumab and pembrolizumab) for first-line treatment of cisplatin-ineligible patients with metastatic UC showed these agents to be feasible and safe [36,37,38].

The anti-PD-1 antibody pembrolizumab was investigated in the KEYNOTE-052 trial for cisplatin-ineligible patients with metastatic disease [37,38]. PD-L1 expression and ORR in the overall cohort were the primary endpoints. The ORR and complete response (CR) rate were observed in 29% and 7% of patients, respectively. Patients with a PD-L1 expression combined positive score (CPS) of ≥10 showed a more prolonged median OS compared to the overall cohort (18.5 months vs. 11.5 months). These results supported the fast track approval of pembrolizumab for cisplatin-ineligible patients in the first-line setting [37,38].

Atezolizumab was approved following IMVigor210, a phase II trial involving 119 treatment-naïve cisplatin-ineligible patients with metastatic UC. The majority of the cohort consisted of patients ineligible for cisplatin due to renal insufficiency and low ECOG performance status; however, a substantial proportion of patients were ineligible due to a history of hearing impairment and peripheral neuropathy [36]. The primary endpoint was ORR and the CPS of PD-L1 expression. Compared to the second-line setting with atezolizumab, a higher ORR was noted in the first-line setting [36]. The U.S. Food and Drug Administration (FDA) approval was granted for atezolizumab in the first-line setting for cisplatin-ineligible patients with locally advanced or metastatic UC (Table 2).

Two trials are investigating the feasibility of a first-line checkpoint inhibition and platinum-based chemotherapy combination after FDA approvals were given for pembrolizumab and atezolizumab for the first- and second-line treatment in cisplatin-ineligible patients.

The efficacy of first-line pembrolizumab with or without chemotherapy (GC or GemCarbo) versus chemotherapy alone for the treatment of metastatic UC is being investigated in a randomized phase III trial (KEYNOTE-361) (Table 3) [43]. IMvigor130 is an ongoing trial of a similar design to investigate the efficacy of atezolizumab (Table 3) [44]. The outcomes of both trials will be stratified by PD-L1 status and cisplatin-eligibility. Early reports have shown that patients with low PD-L1 status in the monotherapy (pembrolizumab or atezolizumab) arms of both trials have inferior survival compared to those with low PD-L1 status in the cisplatin- or carboplatin-based chemotherapy arms. Consequently, the FDA issued a safety alert restricting the use of these immunotherapeutic agents to patients ineligible for cisplatin, patients with low PD-L1 expression tumors, or for patients ineligible for platinum-containing therapy regardless of PD-L1 expression.

### 5.3. Second-Line Immunotherapy for Patients with Locally Advanced or Metastatic Urothelial Cancer

Following the failure of first-line chemotherapy, metastatic UC is a fatal disease with an OS of 6 to 7 months [46]. The application of immune checkpoint inhibitors to the patient population of platinum-refractory UC has modernized the treatment with noteworthy toxicity profiles and durable responses in patients with UC in the second-line setting. Since 2016, five new checkpoint inhibitors targeting PD-1 (nivolumab and pembrolizumab) or PD-L1 (atezolizumab, durvalumab, and avelumab) have gained FDA approval (Table 2) for the treatment of advanced or metastatic UC.

IMvigor210 was a phase II study to assess the activity of the PD-L1 antibody atezolizumab in patients with advanced UC. Results were analyzed according to PD-L1 expression on tumor-infiltrating immune cells (ICs) assessed by immunohistochemistry (Ventana SP142 assay) [47]. The PD-L1 tumor-infiltrating IC status was defined by the ratio of PD-L1 positive immune cells, consequently dividing the tumors into three groups: IC0 (<1%), IC1 (≥1% to <5%) and IC2/3 (≥5%). In patients with platinum-refractory metastatic UC, the IC2/3 subgroup showed higher ORR compared to the counterpart [4]. The FDA approved atezolizumab in 2016 for patients with relapse as a second-line, post-chemotherapy setting.

The recently completed IMvigor211 [48] was an open-label confirmatory phase III randomized trial that compared atezolizumab to the investigator’s choice chemotherapy (taxanes or vinflunine) for platinum-refractory metastatic UC. This study had a hierarchical statistical design to compare differences in OS among the IC2/3 subgroup. However, no significant improvements in OS were shown in the IC2/3 group. Despite failing to meet its primary endpoint, exploratory analysis in the intention to treat population showed durable responses and survival benefits for atezolizumab.

Based on the results from a randomized phase III trial that included patients with platinum-refractory UC (KEYNOTE-045), pembrolizumab was FDA-approved for locally advanced or metastatic UC in the post-chemotherapy setting (Table 2) [42]. The KEYNOTE-045 trial compared OS and progression-free survival (PFS) among the overall cohort and the PD-L1 positive subgroup (CPS ≥10%). CPS was defined as the ratio of the number of PD-L1 expressing cells to the total number of tumor cells [49]. Notably, pembrolizumab prolonged OS regardless of PD-L1 status. In contrast to the IMvigor211 trial in which the chemotherapy arm exhibited better survival outcomes in the PD-L1-high subgroup, the PD-L1-high subgroups of KEYNOTE-045 failed to improve survival in either treatment arm. The data monitoring committee of the study recommended early termination of the trial after it met the primary endpoint for OS [42]. Long-term follow-up data showed that the survival benefit was maintained at 24 months [50].

Nivolumab is a human monoclonal antibody that targets PD-1. The CheckMate 032, a non-randomized phase I/II trial, investigated the feasibility of nivolumab alone for advanced UC patients [51]. The primary endpoints were ORR and progression-free survival, with OS being a secondary endpoint. Treatment with nivolumab provided durable clinical responses in patients with platinum-refractory, locally advanced, or metastatic UC [51]. CheckMate 275 was a single-arm phase II study of nivolumab in patients with platinum-refractory metastatic UC [41]. The study endpoint was ORR in the overall group and the subgroups of patients expressing PD-L1 levels ≥5% and ≥1%. ORRs with a median follow-up of 7 months was 19.6% in the overall population, 28.4% in the PD-L1 ≥5% subgroup, 23.8% in the PD-L1 ≥ 1% subgroup, and 16.1% in the PD-L1-negative subgroup. After observing a significant improvement in OS among all PD-L1 subgroups and the need for an alternative in the second-line setting, the FDA approved nivolumab in 2017 (Table 2).

Study 1108, a single-arm phase I/II study, investigated the efficacy of durvalumab (PD-L1 antibody) in patients with metastatic UC who progressed after platinum-based chemotherapy [40,52]. The study utilized the Ventana SP263 assay for PD-L1 expression, using a cutoff of 25% of PD-L1 positivity on either the tumor cells or the ICs [53]. Responses were observed regardless of PD-L1 expression status. Patients with PD-L1-high tumors achieved a response rate of 46% and a superior OS of 20 months, compared to 8 months in patients with PD-L1-low tumors. Durvalumab was granted FDA approval in 2017 (Table 2).

JAVELIN was a multi-cohort phase Ib trial of avelumab, a human anti-PD-L1 IgG1 antibody, involving patients with metastatic UC who were platinum-refractory or cisplatin-ineligible [39]. Results were analyzed according to PD-L1 status in which the level of positive tumor cells was dichotomized at 5%. The ORR was 40% in the PD-L1-positive subgroup, while 9% was observed in the PD-L1-negative subgroup. Avelumab was granted accelerated FDA approval in 2017 (Table 2).

## 6. Combination Therapy for Locally Advanced and Metastatic Urothelial Carcinoma

Despite promising results of the immune checkpoint inhibitor monotherapy, low long-term durable ORR and high relapse rates warrant alternative approaches for the treatment of metastatic UC. Evidence from studies advocating the immunosuppressive effects of chemotherapy has led to the combination treatment of cytotoxic therapy, which may enhance the efficacy of checkpoint inhibitors [54,55]. However, the results of a phase II, single-arm trial with a combination regimen of GC and ipilimumab failed to achieve its primary endpoint of a one-year OS of >60% [56].

Ongoing trials are continuing to investigate the efficacy of a systemic chemotherapy and checkpoint inhibitor combination in metastatic disease. KEYNOTE-361, a randomized, open-label, phase III trial, compares the efficacies of pembrolizumab, pembrolizumab with or without chemotherapy, and chemotherapy alone for advanced disease. Patients who are cisplatin-eligible will receive GC, and those assigned to chemotherapy who are cisplatin-ineligible will receive GemCarbo. Primary endpoints are OS and PFS, and secondary endpoints are ORR, safety, and tolerability (Table 3) [43]. IMvigor130, a double-blind phase III trial, compares the efficacy and safety between first-line atezolizumab with or without platinum-based chemotherapy and chemotherapy with placebo for locally advanced or metastatic disease (Table 3) [44]. The NILE trial, a randomized phase III trial, investigates first-line durvalumab with or without tremelimumab with standard chemotherapy followed by durvalumab, versus standard of care alone as first-line chemotherapy for advanced or metastatic disease (Table 3) [45]. The primary endpoints are OS and PFS. Secondary endpoints are ORR, duration of response, and time to second progression. Future application of these potential regimens will be determined following the results of the aforementioned clinical trials.

Treatment regimens that combine the inhibitions of the PD-1/PD-L1 and CTLA-4 pathways have the potential for synergistic immunotherapeutic activity. While PD-1 mediates the effector phase of the immune response, the CTLA-4 blockade mediates the priming phase of the immune response. Therefore, the blockage of both immune pathways may enhance anti-tumor activity. The phase I/II CheckMate 032 trial compared the efficacy between either nivolumab or one of two combinations of nivolumab plus ipilimumab in platinum-refractory patients (Table 4) [57]. The combination of 1-mg/kg of nivolumab and 3-mg/kg of ipilimumab provided a higher ORR and CR rate compared to the combination of 3 mg/kg of nivolumab and 1 mg/kg of ipilimumab. Of note, treatment with nivolumab monotherapy resulted in an ORR of 24.4% and a CR rate of 6% [57]. Results from the long-term follow-up of the CheckMate 032 trial reported a higher dosage of ipilimumab to be associated with enhanced antitumor activity without significant toxicities (Table 4) [58]. The combination regimen of 1-mg/kg of nivolumab with 3-mg/kg of ipilimumab provided a higher ORR of 12% and a longer duration of response. Compared to previous reports of PD-1 and PD-L1 monotherapy regimens, a higher ORR and longer PFS and OS rates were achievable with a higher dosage of ipilimumab in the PD-L1 unselected cohort [58]. Based on these results, the combination of a 1-mg/kg of nivolumab and a 3-mg/kg of ipilimumab is being further investigated in the ongoing CheckMate 901 phase III trial, a study of nivolumab with or without ipilimumab versus standard-of-care cisplatin- or carboplatin-based chemotherapy for the management of metastatic UC in the first-line setting (Table 4) [59].

Therapy with durvalumab alone or in combination with tremelimumab is being investigated in a randomized phase II trial (NCT02527434), in which their sequential use is being evaluated regarding safety and efficacy in multiple malignancies. The durvalumab and tremelimumab combination for patients with unresectable stage IV bladder cancer is being evaluated in the DANUBE study, a randomized phase III trial (Table 4) [60]. The primary endpoint is PFS, and the outcomes will be assessed according to PD-L1 status and cisplatin-eligibility.

## 7. Novel Therapeutic Agents for the Treatment of Urothelial Carcinoma

### 7.1. Anti-Angiogenic Therapies

Tumor proliferation is a coordinated result caused by simultaneous activation of the proangiogenic pathways while the immune response is inhibited by the upregulation of immune checkpoint pathways. Tumorigenesis is a process that involves both angiogenesis and immunosuppression in the tumor microenvironment. In theory, simultaneously targeting both pathways may enhance the antitumor capabilities of the immune system [61]. The first study to report data in UC combined a VEGFR-2 antibody, ramucirumab, with the PD-1 antibody pembrolizumab. In a phase I study (Table 5), investigators reported that the combination was well tolerated and showed modest antitumor properties for platinum-refractory metastatic UC [62,63]. A recent phase II study compared the efficacy between docetaxel monotherapy and docetaxel plus ramucirumab or icrucumab in platinum-pretreated patients with locally advanced or metastatic disease [64]. The primary endpoint was unmet; however, PFS was significantly prolonged with the ramucirumab and docetaxel combination [64]. Consistent results were obtained from the subsequent RANGE study, in which a longer PFS was observed with the ramucirumab and docetaxel combination compared to docetaxel-alone (4.1 months vs. 2.8 months) [65]. However, OS outcomes were comparable between both arms [65].

Bevacizumab, a vascular endothelial growth factor (VEGF)-A monoclonal antibody, has shown promising results for metastatic disease. A phase II trial of GC and bevacizumab in the first-line setting of metastatic disease resulted in an OS of 19.1 months and an ORR of 72% [68]. This regimen is being investigated in a phase III trial that compares the efficacy of GC regimen with or without bevacizumab (NCT00942331) [69].

Cabozantinib is a multi-kinase inhibitor that targets *c-MET, RET*, and VEGFR-2, and its combination with checkpoint inhibitors is being investigated [70]. The efficacy of combining cabozantinib with either nivolumab or ipilimumab/nivolumab was evaluated in a phase I trial (Table 5), which provided an ORR of 36% across all genitourinary malignancies [66]. Moreover, additional clinical trials are in progress for the evaluation of combining cabozantinib with atezolizumab (NCT03170960) and pembrolizumab (NCT03534804) [71].

Axitinib, a selective inhibitor of VEGF receptors, is under evaluation for the combined treatment with avelumab in the JAVELIN Medley VEGF trial, a phase II study involving cisplatin-ineligible patients with non-small cell lung carcinoma and metastatic UC in the first-line setting (Table 4) [67]. Lastly, apatinib, a molecule inhibitor of VEGFR-2, is being evaluated for clinical efficacy in combination with pembrolizumab for the treatment of patients with platinum-refractory metastatic UC (NCT03407976) [72].

### 7.2. Gene-Targeted Therapies

Research from previous studies suggests that gene-targeted therapies may accentuate the anti-tumor response of immunotherapy through upregulated immune-mediated killing and inhibition of tumor-mediated immunosuppression [73]. While the majority of fibroblast growth factor receptor (FGFR) mutations are more frequent in non-muscle invasive bladder cancer, alterations in FGFR3 have been observed in particular subtypes of UC. A phase II study of erdafitinib showed an ORR rate of 42% and a CR rate of 3%, with an 80% disease control rate in metastatic chemo-refractory patients with FGFR alterations. Notably, an ORR of 70% was observed in patients treated with checkpoint inhibitors, which is generally a population with a high unmet need for alternative treatment strategies due to poor prognosis [74]. Erdafitinib was FDA-approved in 2019 for patients with FGFR2 and FGFR3-altered advanced UC. At the same time, the first PCR-based diagnostic kit was FDA-approved to detect FGFR alterations in patients with metastatic UC for selecting optimal candidates for erdafitinib administration.

Patients with tumors that have FGFR3 mutation or FGFR fusion are eligible for treatment with AZD4547, an FGFR inhibitor, either as monotherapy or in combination with duvalumab [75]. FORT-2 (NCT03473756; Table 6) is a phase I/II trial of the novel FGFR inhibitor, rogaratinib in combination with atezolizumab [76], and the FIERCE-22 (NCT03123055; Table 6) is a phase I/II trial of pembrolizumab in combination with vofatamab (B-701), a FGFR3 inhibitor [77].

### 7.3. Antibody-Drug Conjugate Therapies

Antibody-drug conjugates (ADC) consist of a protease cleavable linker bound to a cytotoxic agent and a monoclonal antibody that is specific to a highly expressed cancer cell target. The cytotoxic agent is discharged in tumor cells after the lysosomal degradation and internalization of the ADC. ASG-15ME is an ADC that targets SLITRK6, a type I transmembrane neuronal receptor, and has shown positive results in trials regarding UC. SLITRK6 expression is present in 90% of all UCs [78]. In a phase I trial of 51 pretreated patients with metastatic UC who were administered ASG-15ME, results showed an ORR of 37.5% along with 17 (33.3%) partial responses and one (2.0%) CR [79].

Enfortumab vedotin is an ADC comprised of an antibody to nectin-4 bound to a cytotoxic microtubule-disrupting agent, which has shown efficacy in patients with metastatic, platinum-refractory UC. Enfortumab vedotin targets nectin-4, a cell adhesion molecule highly expressed in multiple malignancies, including UC [80]. Enfortumab vedotin was FDA-approved for patients with locally advanced or metastatic disease who have previously received immune checkpoint therapy based on a phase I trial evaluating enfortumab vedotin as a monotherapy. Updated data from a phase I trial evaluating the efficacy of enfortumab vedotin as a second-line treatment in patients with metastatic disease demonstrated an ORR of 40% [81]. Given these impressive outcomes as a monotherapy regimen, a phase I trial (NCT03299545, EV-103) has been initiated to investigate the efficacy of enfortumab vedotin in combination with either atezolizumab or pembrolizumab in both first-line cisplatin-ineligible and platinum-refractory settings [82].

### 7.4. Vaccines

Vaccines may also play a role in immunotherapeutic strategies for UC. Oncoproteins, including human epidermal growth factor receptor 2 (HER2), cancer/testis antigens (CTAs), and tumor-associated antigens, namely, carcinoembryonic antigen (CEA), Mucin-1 (MUC-1), and human chorionic gonadotropin-β (hCG-β), are promising targets. A phase II study (NCT01353222) investigated the therapeutic feasibility of DN24-02 in HER2+ UC patients who are at high risk of relapse following surgery (Table 7). DN24-02 is an autologous immunotherapeutic vaccine that stimulates an immune response against HER2/neu. The vaccine consists of autologous peripheral blood mononuclear cells and APCs that are activated ex vivo with BA7072, a recombinant fusion protein. Results showed that in patients who completed the three infusion cycles, the vaccine increased HER2 antibody responses, serum cytokines (IL-2, IFN-γ, and TNF-α), in vitro IL-2 and IFN-γ accumulation, and antigen-specific T cell responses compared to patients who received standard-of-care surveillance. In subgroup analysis, patients with low tumor burden exhibited more favorable hazard ratios for OS; however, DN24-02 failed to increase OS or recurrence-free survival in the overall group. This study was terminated early due to administrative reasons [83].

CTAs are tumor-associated antigens that elicit a robust immune response and have shown variable expressions in various malignancies. New York esophageal squamous cell cancer 1 (NY-ESO-1) and melanoma-associated antigen-A3 (MAGE A3) are CTAs that are associated with UC. Sharma et al. investigated the feasibility of a recombinant NY-ESO-1 protein vaccine with BCG and GM-CSF as immunological adjuvants in post-cystectomy patients [88]. NY-ESO-1-specific antibody responses were observed, inferring the efficacy of the recombinant NY-ESO-1 protein vaccine to elicit predominant antibody and CD4+ T cell responses. An ongoing phase I clinical trial (NCT01522820) is investigating the safety of NY-ESO-1 vaccine (DEC-205/NY-ESO-1 fusion protein CDX-1401) with or without sirolimus administration in cancer patients with NY-ESO-1 expression (Table 7) [84]. A recently completed non-randomized phase I exploratory study investigated the efficacy of combined intravesical BCG instillation with recombinant MAGE-A3 and immunostimulant AS15 in patients with non-muscle invasive bladder cancer (Table 7) [85]. In half of the patients in that study, intravesical vaccine-specific T cells were increased with a tolerable safety profile. The results inferred that combinations of T-cell vaccines with local immunotherapy may increase total and vaccine-specific T cells in the bladder.

PANVAC is a pox viral cancer vaccine that expresses transgenes for CEA, MUC-1, and T cell costimulatory molecules (B7-1, intracellular adhesion molecule 1, and leukocyte function-associated antigen 3) to boost the anti-tumor T cell immune response. The rationale for this treatment modality is based on studies that have shown increased expression of MUC-1 and CEA in patients with bladder cancer [89,90]. A phase II trial assessed the efficacy of PANVAC with BCG compared to BCG monotherapy in patients with BCG-relapsing non-muscle invasive bladder cancer (Table 7) [86]. Higher rates of tumor-associated antigen response regarding CEA and MUC1 were observed with PANVAC and BCG combination compared to BCG alone.

CDX-1307 is a vaccine that consists of a mannose receptor-specific human monoclonal antibody fused to hCG-β, a tumor antigen frequently expressed in epithelial cancers, including UC [87]. Cytotoxic T-lymphocytes are mediators of antitumor immunity; therefore, a vaccine that induces both cellular and humoral responses to hCG-β is feasible. CDX-1307 presents hCG-β protein to APCs and promotes hCG-β-specific cellular and humoral immune responses. In a phase I trial involving patients with advanced epithelial cancers, the combination of CDX-1307 and immune-stimulating adjuvants induced anti-hCG-β humoral and T cell responses with an acceptable safety profile (Table 7) [87]. Based on these results, a phase II trial (N-ABLE) was initiated to assess the efficacy of CDX-1307 vaccine regimen in patients with hCG-β positive, non-metastatic, muscle-invasive bladder cancer [91]. However, the trial was terminated early due to slow enrollment.

### 7.5. Adoptive T Cell Immunotherapy

Adoptive T cell therapy is a highly custom-tailored cancer therapy that consists of the harvest, proliferation, and modification of human T cells ex vivo, and reinfusion. The promise of adoptive T cell immunotherapy utilizing tumor-infiltrating lymphocytes was based on a study in which more than 50% of patients with advanced melanoma showed a response to treatment [92]. In a clinical study involving patients with metastatic bladder cancer, tumor-reactive lymphocytes were extracted from tumor-draining lymph nodes, followed by in vitro expansion of T lymphocytes against autologous tumor extract, and subsequent reinfusion. In two out of nine patients, durable objective responses were observed without significant adverse effects [93].

In an attempt to broaden the arsenal of adoptive T cell therapy, genetic modification with chimeric antigen receptors (CARs) is in development. CARs consist of antibody-binding domains that are fused to T cell signal domains, allowing a higher affinity binding of target antigens regardless of human leukocyte antigen subtypes [94]. Clinical application of CAR T cells has shown promising antitumor activity in B cell malignancies [95,96]. However, low efficacy and high occurrence of toxicity, which were observed in several trials involving solid tumors, still remain as limitations of CAR T cell immunotherapy [97,98]. Further studies and advancements in genetic engineering to modify CARs and combination strategies with other immunotherapeutics are warranted to overcome the current limitations of adoptive T cell immunotherapy.

## 8. Biomarkers for Predicting Treatment Response

Research and clinical trials are investigating biomarkers that provide information on future responses to cancer immunotherapy [99]. Since patients with advanced UC have low durable response rates, the identification of the patient population likely to benefit from a certain therapy is imperative. Treatment response is associated with multiple factors, including both tumor and immunological factors; therefore, the development of reliable biomarkers is challenging. However, reliable biomarkers may reduce overtreatment in patients without survival benefit and avoid adverse events resulting from treatment.

During the developmental stages of the anti-PD-1/PD-L1 inhibitor, PD-L1 expression on tumor cells or ICs has gained attention as a potential biomarker for checkpoint inhibitors. The Ventana assay used to assess PD-L1 expression in ICs in the cohort 2 of the IMvigor210 trial defined positive PD-L1 as an expression in ≥5% of infiltrating ICs [36,100]. Although the ORR was higher in the PD-L1-positive patients, a 9% response rate was still noted in PD-L1-negative patients. Overall, PD-L1 positivity appears to have a prognostic effect, as shown by the higher OS in the PD-L1-positive subgroup in comparison with the PD-L1-negative subgroup. Nevertheless, in the cisplatin-ineligible cohort 1 of the IMvigor210 trial, PD-L1-negative patients exhibited superior OS in comparison to PD-L1-positive patients [100].

In the KEYNOTE-052 trial, the Dako antibody assay was utilized to define PD-L1 positivity, in which a score of ≥10% was considered positive for PD-L1. Patients with a CPS of ≥10% demonstrated a higher response to pembrolizumab compared to patients with a CPS of <10% [38]. On the other hand, CPS was not associated with ORR in the larger KEYNOTE-045 trial [42]. The different assays used in these trials and the different definitions for PD-L1 positivity are hurdles to defining the exact prognostic significance of PD-L1 expression. Further studies are warranted to institute a uniform method for evaluating PD-L1 expression to predict durable response.

In addition to PD-L1, other biomarkers such as tumor mutation burden (TMB), mismatch repair (MMR) mechanism, and DNA damage response (DDR) and repair pathways are under investigation and have shown promise as potential biomarkers reflecting the response to checkpoint inhibitors in several cancers including UC [101,102,103,104,105]. TMB is the total number of somatic coding mutations found in cancer cells, which represents the deficiency in the mismatch repair mechanism, tumor-infiltrating lymphocytes, neoantigen burden, or immuno-genes signatures. Studies have shown the association of TMB with the tumor response after administration of checkpoint inhibitors in UC and other malignancies. Furthermore, the role of TMB as a biomarker was studied in the post hoc analysis of the IMvigor210 trial, which revealed that patients with a high TMB and luminal cluster II molecular subtypes showed favorable outcomes [36,104]. While further validation is needed, malignancies with MMR defects showed to be responsive to pembrolizumab regardless of the primary source of origin. Based on these results, pembrolizumab was FDA-approved for all malignancies with this specific alteration [102].

Variations in DNA repair pathways are feasible biomarkers for cisplatin sensitivity since such alterations exacerbate the intrinsic cellular DNA repair mechanism. A study has shown that low excision repair cross-complementing 1 (ERCC1) mRNA expressing tumors were associated with prolonged survival [106]. These findings were confirmed in a subsequent meta-analysis of advanced UC patients who underwent platinum-based chemotherapy [105]. A recent study demonstrated that patients with metastatic disease with somatic mutations in DDR proteins to exhibit significantly improved clinical outcomes [107]. Patients with one or more DDR alterations had improved PFS and OS with platinum-based chemotherapy compared to those with no detectable DDR alterations.

## 9. Pseudoprogression and Hyperprogression during Immunotherapy for Urothelial Cancer

Immunotherapy has gained a pivotal role in cancer therapy and has changed the paradigm of advanced UC treatment. However, this represents a challenge to uro-oncologists due to the unique patient responses and side effects resulting from a mechanism of action different from that of conventional chemotherapy. Various clinical trials based on advanced UC have shown that a minority of the patients experience atypical responses to checkpoint inhibitors, namely hyperprogression and pseudoprogression.

Hyperprogression is the rapid paradoxical disease progression after treatment with immunotherapeutic agents, which was first described in patients with melanoma. Champiat et al. reported a prevalence of 6% in patients with multiple solid tumors or lymphoma treated with immunotherapies [108]. The pathogenesis underlying this pattern of clinical progression is still undefined due to the limited number of events reported, the absence of a validated definition, and the apparent absence of a definable biological mechanism [109]. Therefore, results from ongoing clinical trials are needed to confirm the hypothetical mechanisms.

Pseudoprogression is another phenomenon that immunotherapy patients may encounter. The tumor initially increases in size but gradually stabilizes or responds to ongoing treatment. The actual prevalence of such clinical entity in UC is unclear; however, according to retrospective reviews performed by Soria et al., the prevalence of pseudoprogression has an approximate range from 1.5% to 17% [110]. The pathophysiology underlying this phenomenon is considered to be a consequence of the infiltration of ICs within the neoplasm and subsequent temporary increase in volume. A revised radiological criterion to evaluate response in patients treated with immunotherapy has been developed, specifically the immune-response evaluation criteria in solid tumors (iRECIST), which can be utilized to categorize pseudoprogression and other peculiar radiological patterns [111]. The evidence available on this phenomenon is mostly from studies involving other solid malignancies. Therefore, the accumulation of data from UC cohorts is warranted to better define and manage this clinical manifestation associated with immunotherapy.

## 10. Conclusions

Development and research of the PD-1/L1 axis blockade have changed the contemporary treatment paradigm for patients with metastatic UC. The recent approvals of pembrolizumab and atezolizumab for platinum/cisplatin-ineligible patients and checkpoint inhibitors in the second-line setting have redefined the treatment landscape for locally advanced and metastatic UCs. Ongoing clinical trials plan to assess the efficacies of PD-1/L1 antibodies in the first-line setting and to provide further insights into the utilization of PD-L1 expression as a predictive biomarker. Combination immunotherapy regimens, targeted therapies, and antibody-drug conjugates are showing considerable potential as treatment options for UC. The shift in the treatment paradigm, along with the abundance of novel investigational agents, calls for the need to investigate combinations and sequencing of agents in future clinical trials.

## Figures and Tables

**Table 1:** Variant subtypes of urothelial carcinoma.

UC with Divergent Differentiation
With squamous cell differentiation
With glandular differentiation
With trophoblastic differentiation
With small-cell carcinoma
UC with deceptively benign histological features
Nested UC (including large nested)
Microcystic UC
Differential diagnosis with metastases or secondary extension to the bladder
Micropapillary UC
Plasmacytoid/signet ring cell/diffuse UC
Sarcomatoid UC (carcinosarcoma)
Giant cell UC
Clear cell (glycogen-rich) UC
UC, lipid-cell variant
Poorly differentiated tumors (undifferentiated carcinoma NOS, osteoclast-rich undifferentiated carcinoma, undifferentiated carcinoma with rhabdoid features and loss of expression of the SWI/SNF complex
Marked immune cell response
Lymphoepithelioma-like urothelial carcinoma

NOS, not otherwise specified; SWI/SNF, SWItch/sucrose non-fermentable; UC, urothelial carcinoma.

**Table 2:** Current U.S. Food and Drug Administration (FDA) approved immunotherapies for patients with advanced or metastatic urothelial carcinoma.

Agent	FDA Approval	Type	Trial	Indication
Atezolizumab	May 2016	Anti PD-L1	IMvigor 210 [36]	First-line: PD-L1 positive (PD-L1 expression ≥5%) cisplatin-ineligible or platinum-ineligible patients with advanced or metastatic UC Second-line: advanced or metastatic UC following platinum-containing chemotherapy failure
Avelumab	May 2017	Anti PD-L1	JAVELIN [39]	Second-line: advanced or metastatic UC following failure of platinum-based chemotherapy
Durvalumab	May 2017	Anti PD-L1	Study 1108 [40]	Second-line: advanced or metastatic UC following failure of platinum-based chemotherapy
Nivolumab	Feb 2017	Anti PD-1	CheckMate-275 [41]	Second-line: advanced or metastatic UC following failure of platinum-based chemotherapy
Pembrolizumab	May 2017	Anti PD-1	KEYNOTE-045 [42]	First-line: PD-L1 positive (CPS ≥10) cisplatin-ineligible patients with advanced or metastatic UC or patients ineligible for any platinum-based chemotherapy Second-line: advanced or metastatic UC following platinum-containing chemotherapy failure

CPS, combined positive score; PD-1, programmed cell death-1; PD-L1, programmed death-ligand 1; UC, urothelial carcinoma.

**Table 3:** Ongoing trials evaluating a combination of chemotherapy and immunotherapy for urothelial carcinoma.

Combination Agents	Clinical Phase	Identifier	Indication	Primary Endpoints
Pembrolizumab + chemotherapy	III	NCT02853305 (KEYNOTE-361) [43]	First-line: cisplatin-eligible and ineligible patients	PFS and OS
Atezolizumab + gemcitabine + carboplatin/cisplatin	III	NCT02807636 (IMvigor 130) [44]	First-line: locally advanced or metastatic UC	PFS, OS, safety, and tolerability
Durvalumab + gemcitabine + carboplatin/cisplatin	III	NCT03682068 (NILE) [45]	First-line: locally advanced or metastatic UC	PFS and OS

OS: overall survival; PFS: progression-free survival; UC: urothelial carcinoma.

**Table 4:** Ongoing trials evaluating a combination of immunotherapies for urothelial carcinoma.

Combination Agents	Clinical Phase	Identifier	Indication	Primary Endpoints
Ipilimumab + nivolumab	I/II	NCT01928394 (CheckMate 032) [57,58]	Second-line: platinum-refractory advanced UC	ORR
Ipilimumab + nivolumab	III	NCT03036098 (CheckMate 901) [59]	First-line: cisplatin-eligible and ineligible patients	PFS and OS among cisplatin-ineligible patients
Durvalumab + tremelimumab	III	NCT02516241 (DANUBE) [60]	First-line: cisplatin-eligible and ineligible patients	OS among combination arm and PD-L1-high patients in the monotherapy arm

ORR: objective response rate; OS: overall survival; PFS: progression-free survival; UC: urothelial carcinoma.

**Table 5:** Ongoing trials evaluating anti-angiogenic therapies for urothelial carcinoma.

Combination Agents	Clinical Phase	Identifier	Indication	Primary Endpoints
Ramicirumab + pembrolizumab	I	NCT02443324 [62]	Second-line: platinum-refractory advanced UC	Safety
Cabozantinib + nivolumab ± ipilimumab	I	NCT02496208 [66]	Second-line	Safety and toxicity
Axitinib + avelumab	II	NCT03472560 (JAVELIN Medley VEGF) [67]	First-line: cisplatin-ineligible metastatic UC	ORR

ORR: objective response rate; UC: urothelial carcinoma; VEGF: vascular endothelial growth factor.

**Table 6:** Ongoing trials evaluating gene-targeted therapies for urothelial carcinoma.

Combination Agents	Mechanism	Clinical Phase	Identifier	Indication	Primary Endpoints
Rogaratinib + atezolizumab	FGFR target therapy	I/II	NCT03473756 (FORT-2) [76]	First-line: cisplatin-ineligible UC	Toxicity and PFS
Vofatamab + pembrolizumab	FGFR target therapy	I/II	NCT03123055 (FIERCE-22) [77]	Second-line	Safety, toxicity, and ORR

FGFR: fibroblast growth factor receptor; ORR: objective response rate; PFS: progression-free survival; UC: urothelial carcinoma.

**Table 7:** Trials evaluating vaccine therapies for urothelial carcinoma.

Agent	Clinical Phase	Identifier	Indication	Primary Endpoints
DN24-02	II	NCT01353222 [83]	High-risk HER2+ UC with or without prior neoadjuvant chemotherapy	OS
DC205-NY-ESO-1	I	NCT01522820 [84]	Patients with cancer-testis antigen (NY-ESO-1) expressing solid tumors	Safety and toxicity
MAGE-A3 ASCI	I	NCT01498172 [85]	Non-muscle invasive bladder cancer	Adverse events
PANVAC	II	NCT02015104 [86]	BCG-relapsing, high-grade, non-muscle invasive bladder cancer	DFS
CDX-1307	I	NCT00709462 [87]	Incurable bladder cancer	Safety and tolerability

BCG: Bacillus Calmette-Guerin; DFS: disease-free survival; OS: overall survival; UC: urothelial carcinoma.

## References

[1] Siegel R., Miller K.D., Jemal A. (2018). Cancer statistics, 2018. CA Cancer J. Clin..

[2] Chang S.S., Bochner B.H., Chou R., Dreicer R., Kamat A.M., Lerner S.P., Lotan Y., Meeks J.J., Michalski J.M., Morgan T.M. (2017). Treatment of Non-Metastatic Muscle-Invasive Bladder Cancer: AUA/ASCO/ASTRO/SUO Guideline. J. Urol..

[3] Spiess P.E., Agarwal N., Bangs R., Boorjian S.A., Buyyounouski M.K., Clark P.E., Downs T.M., Efstathiou J.A., Flaig T.W., Friedlander T. (2017). Bladder cancer, version 5. 2017, NCCN Clinical Practice Guidelines in Oncology. J. Natl. Compr. Cancer Netw..

[4] Rosenberg J.E., Hoffman-Censits J., Powles T., van der Heijden M.S., Balar A.V., Necchi A., Dawson N., O’Donnell P.H., Balmanoukian A., Loriot Y. (2016). Atezolizumab in patients with locally advanced and metastatic urothelial carcinoma who have progressed following treatment with platinum-based chemotherapy: A single-arm, multicentre, phase 2 trial. Lancet.

[5] Lopez-Beltran A., Henriques V., Montironi R., Cimadamore A., Raspollini M.R., Cheng L. (2019). Variants and new entities of bladder cancer. Histopathology.

[6] Moch H., Humphrey P.A., Ulbright T.M., Reuter V. (2016). WHO Classification of Tumours of the Urinary System and Male Genital Organs.

[7] Kim S.P., Frank I., Cheville J.C., Thompson R.H., Weight C.J., Thapa P., Boorjian S.A. (2012). The impact of squamous and glandular differentiation on survival after radical cystectomy for urothelial carcinoma. J. Urol..

[8] Scosyrev E., Ely B.W., Messing E.M., Speights V.O., Grossman H.B., Wood D.P., de Vere White R.W., Vogelzang N.J., Trump D.L., Natale R.B. (2011). Do mixed histological features affect survival benefit from neoadjuvant platinum-based combination chemotherapy in patients with locally advanced bladder cancer? A secondary analysis of Southwest Oncology Group- Directed Intergroup Study (S8710). BJU Int..

[9] Tamas E.F., Nielsen M.E., Schoenberg M.P., Epstein J.I. (2007). Lymphoepithelioma-like carcinoma of the urinary tract: A clinicopathological study of 30 pure and mixed cases. Mod. Pathol..

[10] Lopez-Beltran A., Paner G., Blanca A., Montironi R., Tsuzuki T., Nagashima Y., Chuang S.S., Win K.T., Madruga L., Raspollini M.R. (2017). Lymphoepithelioma-like carcinoma of the upper urinary tract. Virchows Arch..

[11] Robertson A.G., Kim J., Al-Ahmadie H., Bellmunt J., Guo G., Cherniack A.D., Hinoue T., Laird P.W., Hoadley K.A., Akbani R. (2017). Comprehensive Molecular Characterization of Muscle-Invasive Bladder Cancer. Cell.

[12] Roupret M., Babjuk M., Comperat E., Zigeuner R., Sylvester R.J., Burger M., Cowan N.C., Böhle A., Van Rhijn B.W., Kaasinen E. (2015). European Association of Urology guidelines on upper urinary tract urothelial cell carcinoma: 2015 update. Eur. Urol..

[13] Galsky M.D., Hahn N.M., Rosenberg J., Sonpavde G., Hutson T., Oh W.K., Dreicer R., Vogelzang N., Sternberg C.N., Bajorin D.F. (2011). Treatment of patients with metastatic urothelial cancer “unfit” for cisplatin-based chemotherapy. J. Clin. Oncol..

[14] Kaufman D., Raghavan D., Carducci M., Levine E.G., Murphy B., Aisner J., Kuzel T., Nicol S., Oh W., Stadler W. (2000). Phase II trial of gemcitabine plus cisplatin in patients with metastatic urothelial cancer. J. Clin. Oncol..

[15] von der Maase H., Hansen S.W., Roberts J.T., Dogliotti L., Oliver T., Moore M.J., Bodrogi I., Albers P., Knuth A., Lippert C.M. (2000). Gemcitabine and cisplatin versus methotrexate, vinblastine, doxorubicin, and cisplatin in advanced or metastatic bladder cancer: Results of a large, randomized, multinational, multicenter, phase III study. J. Clin. Oncol..

[16] McCaffrey J.A., Hilton S., Mazumdar M., Sadan S., Kelly W.K., Scher H.I., Bajorin D.F. (1997). Phase II trial of docetaxel in patients with advanced or metastatic transitional-cell carcinoma. J. Clin. Oncol..

[17] Sweeney C.J., Roth B.J., Kabbinavar F.F., Vaughn D.J., Arning M., Curiel R.E., Obasaju C.K., Wang Y., Nicol S.J., Kaufman D.S. (2006). Phase II study of pemetrexed for second-line treatment of transitional cell cancer of the urothelium. J. Clin. Oncol..

[18] Sternberg C.N., Yagoda A., Scher H.I., Watson R.C., Geller N., Herr H.W., Morse M.J., Sogani P.C., Vaughan E.D., Bander N. (1989). Methotrexate, vinblastine, doxorubicin, and cisplatin for advanced transitional cell carcinoma of the urothelium. Efficacy and patterns of response and relapse. Cancer.

[19] Loehrer P.J., Einhorn L.H., Elson P.J., Crawford E.D., Kuebler P., Tannock I., Raghavan D., Stuart-Harris R., Sarosdy M.F., Lowe B.A. (1992). A randomized comparison of cisplatin alone or in combination with methotrexate, vinblastine, and doxorubicin in patients with metastatic urothelial carcinoma: A cooperative group study. J. Clin. Oncol..

[20] von der Maase H., Sengelov L., Roberts J.T., Ricci S., Dogliotti L., Oliver T., Moore M.J., Zimmermann A., Arning M. (2005). Long-term survival results of a randomized trial comparing gemcitabine plus cisplatin with methotrexate, vinblastine, doxorubicin, plus cisplatin in patients with bladder cancer. J. Clin. Oncol..

[21] Bellmunt J., von der Maase H., Mead G.M., Skoneczna I., De Santis M., Daugaard G., Boehle A., Chevreau C., Paz-Ares L., Laufman L.R. (2012). Randomized phase III study comparing paclitaxel/cisplatin/gemcitabine and gemcitabine/cisplatin in patients with locally advanced or metastatic urothelial cancer without prior systemic therapy: EORTC Intergroup Study 30987. J. Clin. Oncol..

[22] Dash A., Galsky M.D., Vickers A.J., Serio A.M., Koppie T.M., Dalbagni G., Bochner B.H. (2006). Impact of renal impairment on eligibility for adjuvant cisplatin-based chemotherapy in patients with urothelial carcinoma of the bladder. Cancer.

[23] De Santis M., Bellmunt J., Mead G., Kerst J.M., Leahy M., Maroto P., Gil T., Marreaud S., Daugaard G., Skoneczna I. (2012). Randomized phase II/III trial assessing gemcitabine/carboplatin and methotrexate/ carboplatin/vinblastine in patients with advanced urothelial cancer who are unfit for cisplatin-based chemotherapy: EORTC study 30986. J. Clin. Oncol..

[24] Hainsworth J.D., Meluch A.A., Litchy S., Schnell F.M., Bearden J.D., Yost K., Greco F.A. (2005). Paclitaxel, carboplatin, and gemcitabine in the treatment of patients with advanced transitional cell carcinoma of the urothelium. Cancer.

[25] Hurwitz M.E., Sokhn J., Petrylak D.P. (2016). Cancer immunotherapy: New applications in urologic oncology. Curr. Opin. Urol..

[26] Donin N.M., Lenis A.T., Holden S., Drakaki A., Pantuck A., Belldegrun A., Chamie K. (2017). Immunotherapy for the treatment of urothelial carcinoma. J. Urol..

[27] Anderson A.C., Anderson D.E., Bregoli L., Hastings W.D., Kassam N., Lei C., Chandwaskar R., Karman J., Su E.W., Hirashima M. (2007). Promotion of tissue inflammation by the immune receptor Tim-3 expressed on innate immune cells. Science.

[28] Schepisi G., Brighi N., Cursano M.C., Gurioli G., Ravaglia G., Altavilla A., Burgio S.L., Testoni S., Menna C., Farolfi A. (2019). Inflammatory biomarkers as predictors of response to immunotherapy in urological tumors. J. Oncol..

[29] Sica G.L., Choi I.H., Zhu G., Tamada K., Wang S.D., Tamura H., Chapoval A.I., Flies D.B., Bajorath J., Chen L. (2003). B7-H4, a molecule of the B7 family, negatively regulates T cell immunity. Immunity.

[30] Loos M., Hedderich D.M., Friess H., Kleeff J. (2010). B7-H3 and its role in antitumor immunity. Clin. Dev. Immunol..

[31] Le D.T., Uram J.N., Wang H., Bartlett B.R., Kemberling H., Eyring A.D., Skora A.D., Luber B.S., Azad N.S., Laheru D. (2015). PD-1 blockade in tumors with mismatch-repair deficiency. N. Engl. J. Med..

[32] Kelderman S., Schumacher T.N., Kvistborg P. (2015). Mismatch repair-deficient cancers are targets for anti-PD-1 therapy. Cancer Cell.

[33] Singh P., Black P. (2016). Emerging role of checkpoint inhibition in localized bladder cancer. Urologic Oncology: Seminars and Original Investigations.

[34] Lawrence M.S., Stojanov P., Polak P., Kryukov G.V., Cibulskis K., Sivachenko A., Carter S.L., Stewart C., Mermel C.H., Roberts S.A. (2013). Mutational heterogeneity in cancer and the search for new cancer-associated genes. Nature.

[35] Alexandrov L.B., Nik-Zainal S., Wedge D.C., Aparicio S.A., Behjati S., Biankin A.V., Bignell G.R., Bolli N., Borg A., Børresen-Dale A.L. (2013). Signatures of mutational processes in human cancer. Nature.

[36] Balar A.V., Galsky M.D., Rosenberg J.E., Powles T., Petrylak D.P., Bellmunt J., Loriot Y., Necchi A., Hoffman-Censits J., Perez-Gracia J.L. (2017). Atezolizumab as first-line treatment in cisplatin ineligible patients with locally advanced and metastatic urothelial carcinoma: A single-arm, multicentre, phase 2 trial. Lancet.

[37] O’Donnel P., Grivas P., Balar A.V., Bellmunt J., Vuky J., Powles T., Plimack E.R., Hahn N.M., De Wit R., Pang L. Biomarker findings and mature clinical results from KEYNOTE-052: First-line pembrolizumab (pembro) in cisplatin-ineligible advanced urothelial cancer (UC). Proceedings of the 2017 ASCO Annual Meeting Genitourinary (Nonprostate) Cancer Oral Abstract Session.

[38] Balar A.V., Castellano D., O’Donnell P.H., Grivas P., Vuky J., Powles T., Plimack E.R., Hahn N.M., de Wit R., Pang L. (2017). First-line pembrolizumab in cisplatin-ineligible patients with locally advanced and unresectable or metastatic urothelial cancer (KEYNOTE-052): A multicentre, single-arm, phase 2 study. Lancet Oncol..

[39] Patel M.R., Ellerton J., Infante J.R., Agrawal M., Gordon M., Aljumaily R., Britten C.D., Dirix L., Lee K.W., Taylor M. (2018). Avelumab in metastatic urothelial carcinoma after platinum failure (JAVELIN Solid Tumor): Pooled results from two expansion cohorts of an open-label, phase 1 trial. Lancet Oncol..

[40] Powles T., O’Donnell P.H., Massard C., Arkenau H.T., Friedlander T.W., Hoimes C.J., Lee J.L., Ong M., Sridhar S.S., Vogelzang N.J. (2017). Efficacy and safety of durvalumab in locally advanced or metastatic urothelial carcinoma: Updated results from a phase 1/2 open-label study. JAMA Oncol..

[41] Sharma P., Retz M., Siefker-Radtke A., Baron A., Necchi A., Bedke J., Plimack E.R., Vaena D., Grimm M.O., Bracarda S. (2017). Nivolumab in metastatic urothelial carcinoma after platinum therapy (CheckMate 275): A multicentre, single-arm, phase 2 trial. Lancet Oncol..

[42] Bellmunt J., de Wit R., Vaughn D.J., Fradet Y., Lee J.L., Fong L., Vogelzang N.J., Climent M.A., Petrylak D.P., Choueiri T.K. (2017). Pembrolizumab as second-line therapy for advanced urothelial carcinoma. N. Engl. J. Med..

[43] Powles T., Gschwend J.E., Loriot Y., Bellmunt J., Geczi L., Vulsteke C., Abdelsalam M., Gafanov R., Bae W.K., Revesz J. (2017). Phase 3 KEYNOTE-361 trial: Pembrolizumab (pembro) with or without chemotherapy versus chemotherapy alone in advanced urothelial cancer. J. Clin. Oncol..

[44] Galsky M.D., Grande E., Davis I.D., Santis M.D., Arija J.A.A., Kikuchi E., Mecke A., Thastrom A.C., Bamias A. (2018). IMvigor130: A randomized, phase III study evaluating first-line (1L) atezolizumab (atezo) as monotherapy and in combination with platinum-based chemotherapy (chemo) in patients (pts) with locally advanced or metastatic urothelial carcinoma (mUC). J. Clin. Oncol..

[45] Galsky M.D., Necchi A., Sridhar S.S., Ogawa O., Angra N., Hois S., He P., Ghiorghiu D.C., Bellmunt J. (2019). A phase III, randomized, open label, multicenter, global study of first-line (1L) durvalumab in combination with standard of care (SOC) chemotherapy and durvalumab in combination with tremelimumab and SOC chemotherapy versus SOC chemotherapy alone in patients with unresectable locally advanced or metastatic urothelial cancer (UC). J. Clin. Oncol..

[46] Bellmunt J., Theodore C., Demkov T., Komyakov B., Sengelov L., Daugaard G., Caty A., Carles J., Jagiello-Gruszfeld A., Karyakin O. (2009). Phase III trial of vinflunine plus best supportive care compared with best supportive care alone after a platinum containing regimen in patients with advanced transitional cell carcinoma of the urothelial tract. J. Clin. Oncol..

[47] Herbst R.S., Soria J.C., Kowanetz M., Fine G.D., Hamid O., Gordon M.S., Sosman J.A., McDermott D.F., Powderly J.D., Gettinger S.N. (2014). Predictive correlates of response to the anti-PD-L1 antibody MPDL3280A in cancer patients. Nature.

[48] Powles T., Duran I., van der Heijden M.S., Loriot Y., Vogelzang N.J., De Giorgi U., Oudard S., Retz M.M., Castellano D., Bamias A. (2018). Atezolizumab versus chemotherapy in patients with platinum-treated locally advanced or metastatic urothelial carcinoma (IMvigor211): A multicentre, open-label, phase 3 randomised controlled trial. Lancet.

[49] Balar A.V., Bellmunt J., O’Donnell P.H., Castellano D., Grivas P., Vuky J., Powles T., Plimack E.R., Hahn N.M., de Wit R. (2016). Pembrolizumab (pembro) as first-line therapy for advanced/unresectable or metastatic urothelial cancer: Preliminary results from the phase 2 KEYNOTE-052 study. Ann. Oncol..

[50] Bellmunt J., De Wit R., Vaughn D.J., Fradet Y., Lee J., Fong L., Vogelzang N.J., Climent M.A., Petrylak D.P., Choueiri T.K. (2018). Two-year follow up from the phase 3 KEYNOTE-045 trial of pembrolizumab (pembro) vs investigator’s choice (paclitaxel, docetaxel, or vinflunine) in recurrent, advanced urothelial cancer (UC). J. Clin. Oncol..

[51] Sharma P., Callahan M.K., Bono P., Kim J., Spiliopoulou P., Calvo E., Pillai R.N., Ott P.A., de Braud F., Morse M. (2016). Nivolumab monotherapy in recurrent metastatic urothelial carcinoma (CheckMate 032): A multicentre, open-label, two-stage, multi-arm, phase 1/2 trial. Lancet Oncol..

[52] Massard C., Gordon M.S., Sharma S., Rafii S., Wainberg Z.A., Luke J., Curiel T.J., Colon-Otero G., Hamid O., Sanborn R.E. (2016). Safety and efficacy of durvalumab (MEDI4736), an anti-programmed cell death ligand-1 immune checkpoint inhibitor, in patients with advanced urothelial bladder cancer. J. Clin. Oncol..

[53] Marlon Rebelatto A.M., Sabalos C., Walker J., Midha A., Steele K., Robbins P.B., Li X., Shi L., Blake-Haskins J.A., Ibrahim R.A. (2015). Development of a PD-L1 companion diagnostic assay for treatment with MEDI4736 in NSCLC and SCCHN patients. J. Clin. Oncol..

[54] Chang C.H., Qiu J., O’Sullivan D., Buck M.D., Noguchi T., Curtis J.D., Chen Q., Gindin M., Gubin M.M., van der Windt G.J. (2015). Metabolic competition in the tumor microenvironment is a driver of cancer progression. Cell.

[55] Hato S.V., Khong A., de Vries I.J., Lesterhuis W.J. (2014). Molecular pathways: The immunogenic effects of platinum-based chemotherapeutics. Clin. Cancer Res..

[56] Galsky M.D., Wang H., Hahn N.M., Twardowski P., Pal S.K., Albany C., Fleming M.T., Starodub A., Hauke R.J., Yu M. (2018). Phase 2 trial of gemcitabine, cisplatin, plus ipilimumab in patients with metastatic urothelial cancer and impact of DNA damage response gene mutations on outcomes. Eur. Urol..

[57] Sharma P., Callahan M.K., Calvo A. Efficacy and safety of nivolumab plus ipilimumab in previously treated metastatic urothelial carcinoma: First results from the phase I/II CheckMate 032 study. Proceedings of the 2016 SITC Annual Meeting, National Harbor.

[58] Rosenberg J., Sharma P., De Braud F., Basso U., Calvo E., Bono P., Morse M., Ascierto P.A., Lopez-Martin J.A., Brossart P. (2018). Nivolumab(N) alone or in combination with ipilimumab (I) in patients (pts) with platinum-pretreated metastatic urothelial carcinoma (mUC), including the nivolumab 1 mg/kg + ipilimumab 3 mg/kg expansion from CheckMate 032. Ann. Oncol..

[59] Galsky M.D., Powles T., Li S., Hennicken D., Sonpavde G. (2018). A phase 3, open-label, randomized study of nivolumab plus ipilimumab or standard of care (SoC) vs SoC alone in patients (pts) with previously untreated unresectable or metastatic urothelial carcinoma (mUC.; CheckMate 901). J. Clin. Oncol..

[60] Powles T., Galsky M.D., Castellano D., Van Der Heijden M.S., Petrylak D.P., Armstrong J., Belli R., Ferro S., Ben. Y., Bellmunt J. (2016). A phase 3 study of first-line durvalumab (MEDI4736) ± tremelimumab versus standard of care (SoC) chemotherapy(CT) in patients (pts) with unresectable stage IV urothelial bladder cancer (UBC): DANUBE. J. Clin. Oncol..

[61] Motz G.T., Coukos G. (2011). The parallel lives of angiogenesis and immunosuppression: Cancer and other tales. Nat. Rev. Immunol..

[62] Petrylak D.P., Arkenau H.-T., Perez-Gracia J.L., Krebs M., Santana- Davila R., Yang J., Rege J., Mi G., Ferry D., Herbst R.S. (2017). A multicohort phase I study of ramucirumab (R) plus pembrolizumab (P): Interim safety and clinical activity in patients with urothelial carcinoma. J. Clin. Oncol..

[63] Herbst R.S., Chau I., Petrylak D.P., Arkenau H.-T., Bendell J.C., Santana-Davila R., Calvo E., Penel N., Martin-Liberal J., Soriano A.O. (2018). Activity of ramucirumab (R) with pembrolizumab(P) by PD-L1 expression in advanced solid tumors: Phase 1a/b study in later lines of therapy. J. Clin. Oncol..

[64] Petrylak D.P., Tagawa S.T., Kohli M., Eisen A., Canil C., Sridhar S.S., Spira A., Yu E.Y., Burke J.M., Shaffer D. (2016). Docetaxel as monotherapy or combined with ramucirumab or icrucumab in second-line treatment for locally advanced or metastatic urothelial carcinoma: An open-label, three arm, randomized controlled phase II trial. J. Clin. Oncol..

[65] Petrylak D.P., de Wit R., Chi K.N., Drakaki A., Sternberg C.N., Nishiyama H., Castellano D., Hussain S., Fléchon A., Bamias A. (2017). Ramucirumab plus docetaxel versus placebo plus docetaxel in patients with locally advanced or metastatic urothelial carcinoma after platinum-based therapy (RANGE): A randomised, double-blind, phase 3 trial. Lancet.

[66] Apolo A.B., Mortazavi A., Stein M.N., Davarpanah N.N., Nadal R.M., Parnes H.L., Ning Y.M., Francis D.C., Cordes L.M., Berniger M.A. (2017). A phase I study of cabozantinib plus nivolumab (CaboNivo) and cabonivo plus ipilimumab (CaboNivoIpi) in patients (pts) with refractory metastatic (m) urothelial carcinoma (UC) and other genitourinary (GU) tumors. J. Clin. Oncol..

[67] US National Library of Medicine A Study of Avelumab in Combination with Axitinib in Non-Small Cell Lung Cancer (NSCLC) or Urothelial Cancer (Javelin Medley VEGF). https://clinicaltrials.gov/ct2/show/NCT03472560.

[68] Hahn N.M., Stadler W.M., Zon R.T., Waterhouse D., Picus J., Nattam S., Johnson C.S., Perkins S.M., Waddell M.J., Sweeney C.J. (2011). Phase II trial of cisplatin, gemcitabine, and bevacizumab as first-line therapy for metastatic urothelial carcinoma: Hoosier Oncology Group GU 04-75. J. Clin. Oncol..

[69] US National Library of Medicine Gemcitabine Hydrochloride and Cisplatin with or Without Bevacizumab in Treating Patients with Advanced Urinary Tract Cancer. https://clinicaltrials.gov/ct2/show/NCT00942331.

[70] Yakes F.M., Chen J., Tan J., Yamaguchi K., Shi Y.C., Yu P.W., Qian F., Chu F., Bentzien F., Cancilla B. (2011). Cabozantinib (XL184), a novel MET and VEGFR2 inhibitor, simultaneously suppresses metastasis, angiogenesis, and tumor growth. Mol. Cancer Ther..

[71] Maia M.C., Agarwal N., McGregor B.A., Vaishampayan U.N., Choueiri T.K., Green M.C. (2018). Phase 1b trial of cabozantinib in combination with atezolizumab in patients with locally advanced or metastatic urothelial carcinoma (UC) or renal cell carcinoma (RCC). J. Clin. Oncol..

[72] US National Library of Medicine Apatinib with Pembrolizumab in Previously Treated Advanced Malignancies (APPEASE). https://clinicaltrials.gov/ct2/show/NCT03407976.

[73] Vanneman M., Dranoff G. (2012). Combining immunotherapy and targeted therapies in cancer treatment. Nat. Rev. Cancer.

[74] Siefker-Radtke A.O., Necchi A., Park S.H., GarcÃa-Donas J., Huddart R.A., Burgess E.F., Fleming M.T., Rezazadeh A., Mellado B., Varlamov S. (2018). First results from the primary analysis population of the phase 2 study of erdafitinib (ERDA.; JNJ-42756493) in patients (pts) with metastatic or unresectable urothelial carcinoma (mUC) and FGFR alterations (FGFRalt). J. Clin. Oncol..

[75] Gavine P.R., Mooney L., Kilgour E., Thomas A.P., Al-Kadhimi K., Beck S., Rooney C., Coleman T., Baker D., Mellor M.J. (2012). AZD4547: An orally bioavailable, potent, and selective inhibitor of the fibroblast growth factor receptor tyrosine kinase family. Cancer Res..

[76] Joerger M., Cassier P., Penel N., Cathomas R., Richly H., Schostak M. (2018). Rogaratinib treatment of patients with advanced urothelial carcinomas prescreened for tumor FGFR mRNA expression. J. Clin. Oncol..

[77] Siefker-Radtke A.O., Currie G., Abella E., Vaena D.A., Kalebasty A.R., Curigliano G., Tupikowski K., Andric Z.G., Lugowska I., Kelly W.K. (2019). Clinical activity of vofatamab (V) a FGFR3 selective inhibitor in combination with pembrolizumab (P) in WT metastatic urothelial carcinoma, preliminary analysis. J. Clin. Oncol..

[78] Morrison K., Challita-Eid P.M., Raitano A., An Z., Yang P., Abad J.D., Liu W., Lortie D.R., Snyder J.T., Capo L. (2016). Development of ASG-15ME, a novel antibody-drug conjugate targeting SLITRK6, a new urothelial cancer biomarker. Mol. Cancer Ther..

[79] Petrylak D.P., Heath E., Sonpavde G., George S., Morgans A., Eigl B.J., Picus J., Cheng S., Hotte S.J., Gartner E. (2016). Interim analysis of a phase 1 dose escalation trial of the antibody drug conjugate (ADC) AGS15E (ASG-15ME) in patients (Pts) with metastatic urothelial cancer (mUC). Ann. Oncol..

[80] Petrylak D.P., Perez R., Zhang J., Smith D., Ruether J., Sridhar S.S., Sangha R.S., Lang J.M., Heath E.I., Merchan J.R. (2017). A phase I study of enfortumab vedotin (ASG-22CE.; ASG-22ME): Updated analysis of patients with metastatic urothelial cancer. J. Clin. Oncol..

[81] Rosenberg J., Sridhar S.S., Zhang J., Smith D., Ruether J., Flaig T., Baranda J.C., Lang J.M., Plimack E.R., Sangha R.S. (2018). Updated results from the enfortumab vedotin phase 1 (EV-101) study in patients with metastatic urothelial cancer (mUC). J. Clin. Oncol..

[82] Hoimes C.J., Petrylak D.P., Flaig T.W., Carret A.S., Melhem-Bertrandt A., Rosenberg J.E. (2018). EV-103 study: A phase 1b dose-escalation and dose expansion study of enfortumab vedotin in combination with immune checkpoint inhibitor (CPI) therapy for treatment of patients with locally advanced or metastatic urothelial cancer. J. Clin. Oncol..

[83] Bajorin D.F., Sharma P., Quinn D.I., Plimack E.R. (2016). Phase 2 trial results of DN24-02, a HER2-targeted autologous cellular immunotherapy in HER2+urothelial cancer patients (pts). J. Clin. Oncol..

[84] US National Library of Medicine Vaccine Therapy with or without Sirolimus in Treating Patients with NY-ESO-1 Expressing Solid tumors. https://clinicaltrials.gov/ct2/show/NCT01522820.

[85] Derré L., Cesson V., Lucca I., Cerantola Y., Valerio M., Fritschi U., Vlamopoulos Y., Burruni R., Legris A.S., Dartiguenave F. (2017). Intravesical Bacillus Calmette Guerin combined with a cancer-vaccine increases local T-cell responses in non-muscle-invasive bladder cancer patients. Clin. Cancer Res..

[86] Sanford T., Donahue R., Jochems C., Dolan R., Bellfield S., Anderson M., Singer E., Weiss R., Elsamra S., Jang T. (2017). Immunologic response to a therapeutic cancer vaccine (PANVAC): Initial results from a randomized phase 2 clinical trial (abstract MP15-10). J. Urol..

[87] Morse M.A., Chapman R., Powderly J., Blackwell K., Keler T., Green J., Riggs R., He L.Z., Ramakrishna V., Vitale L. (2011). Phase I study utilizing a novel antigen-presenting cell-targeted vaccine with toll-like receptor stimulation to induce immunity to self-antigens in cancer patients. Clin. Cancer Res..

[88] Sharma P., Bajorin D., Jungbluth A., Herr H., Old L., Gnjatic S. (2008). Immune responses detected in urothelial carcinoma patients after vaccination with NY-ESO-1 protein plus BCG and GM-CSF. J. Immunother..

[89] Ahmad S., Lam T.B., N’Dow J. (2015). Significance of MUC1 in bladder cancer. BJU Int..

[90] D’Costa J.J., Goldsmith J.C., Wilson J.S., Bryan R.T., Ward D.G. (2016). A systematic review of the diagnostic and prognostic value of urinary protein biomarkers in urothelial bladder cancer. Bladder Cancer.

[91] US National Library of Medicine A Study of the CDX-1307 Vaccine Regimen in Patients With Newly Diagnosed Muscle-Invasive Bladder Cancer (The "N-ABLE" Study). https://clinicaltrials.gov/ct2/show/NCT01094496.

[92] Dudley M.E., Wunderlich J.R., Yang J.C., Sherry R.M., Topalian S.L., Restifo N.P., Royal R.E., Kammula U., White D.E., Mavroukakis S.A. (2005). Adoptive cell transfer therapy following non-myeloablative but lymphodepleting chemotherapy for the treatment of patients with refractory metastatic melanoma. J. Clin. Oncol..

[93] Sherif A., Hasan M.N., Radecka E., Rodriguez A.L., Shabo S., Karlsson M., Schumacher M.C., Martis P., Winqvist O. (2015). Pilot study of adoptive immunotherapy with sentinel node- derived Tcells in muscle-invasive urinary bladder cancer. Scand. J. Urol..

[94] Maude S.L., Frey N., Shaw P.A., Aplenc R., Barrett D.M., Bunin N.J., Chew A., Gonzalez V.E., Zheng Z., Lacey S.F. (2014). Chimeric antigen receptor T cells for sustained remissions in leukemia. N. Engl. J. Med..

[95] Porter D.L., Levine B.L., Kalos M., Bagg A., June C.H. (2011). Chimeric antigen receptor-modified T cells in chronic lymphoid leukemia. N. Engl. J. Med..

[96] Grupp S.A., Kalos M., Barrett D., Aplenc R., Porter D.L., Rheingold S.R., Teachey D.T., Chew A., Hauck B., Wright J.F. (2013). Chimeric antigen receptor-modified T cells for acute lymphoid leukemia. N. Engl. J. Med..

[97] Lamers C.H., Sleijfer S., van Steenbergen S., van Elzakker P., van Krimpen B., Groot C., Vulto A., den Bakker M., Oosterwijk E., Debets R. (2013). Treatment of metastatic renal cell carcinoma with CAIX CAR-engineered Tcells: Clinical evaluation and management of on-target toxicity. Mol. Ther..

[98] Morgan R.A., Yang J.C., Kitano M., Dudley M.E., Laurencot C.M., Rosenberg S.A. (2010). Case report of a serious adverse event following the administration of T cells transduced with a chimeric antigen receptor recognizing ERBB2. Mol. Ther..

[99] Kitano S., Nakayama T., Yamashita M. (2018). Biomarkers for immune checkpoint inhibitors in melanoma. Front. Oncol..

[100] Balar A.V., Loriot Y., Perez-Gracia J.L., Hoffman-Censits J.H., Petrylak D.P., Van Der Heijden M.S., Ding B., Shen X., Rosenberg J.E. (2018). Atezolizumab (atezo) in first-line cisplatin-ineligible or platinum-treated locally advanced or metastatic urothelial cancer (mUC): Long-term efficacy from phase 2 study IMvigor210. J. Clin. Oncol..

[101] Rizvi N.A., Hellmann M.D., Snyder A., Kvistborg P., Makarov V., Havel J.J., Lee W., Yuan J., Wong P., Ho T.S. (2015). Cancer immunology. Mutational landscape determines sensitivity to PD-1 blockade in non-small cell lung cancer. Science.

[102] Le D.T., Durham J.N., Smith K.N., Wang H., Bartlett B.R., Aulakh L.K., Lu S., Kemberling H., Wilt C., Luber B.S. (2017). Mismatch repair deficiency predicts response of solid tumors to PD-1 blockade. Science.

[103] Havel J.J., Chowell D., Chan T.A. (2019). The evolving landscape of biomarkers for checkpoint inhibitor immunotherapy. Nat. Rev. Cancer.

[104] Chan T.A., Yarchoan M., Jaffee E., Swanton C., Quezada S.A., Stenzinger A., Peters S. (2019). Development of tumor mutation burden as an immunotherapy biomarker: Utility for the oncology clinic. Ann. Oncol..

[105] Urun Y., Leow J.J., Fay A.P., Albiges L., Choueiri T.K., Bellmunt J. (2017). ERCC1 as a prognostic factor for survival in patients with advanced urothelial cancer treated with platinum based chemotherapy: A systematic review and meta-analysis. Crit. Rev. Oncol. Hematol..

[106] Bellmunt J., Paz-Ares L., Cuello M., Cecere F.L., Albiol S., Guillem V., Gallardo E., Carles J., Mendez P., de la Cruz J.J. (2007). Spanish Oncology Genitourinary Group. Gene expression of ERCC1 as a novel prognostic marker in advanced bladder cancer patients receiving cisplatin-based chemotherapy. Ann. Oncol..

[107] Teo M.Y., Bambury R.M., Zabor E.C., Jordan E., Al-Ahmadie H., Boyd M.E., Bouvier N., Mullane S.A., Cha E.K., Roper N. (2017). DNA damage response and repair gene alterations are associated with improved survival in patients with platinum-treated advanced urothelial carcinoma. Clin. Cancer Res..

[108] Champiat S., Dercle L., Ammari S., Massard C., Hollebecque A., Postel-Vinay S., Chaput N., Eggermont A., Marabelle A., Soria J.C. (2017). Hyperprogressive disease is a new pattern of progression in cancer patients treated by anti-PD-1/PD-L1. Clin. Cancer Res..

[109] Wang Q., Gao J., Wu X. (2018). Pseudoprogression and hyperprogression after checkpoint blockade. Int. Immunopharmacol..

[110] Soria F., Beleni A.I., D’Andrea D., Resch I., Gust K.M., Gontero P., Shariat S.F. (2018). Pseudoprogression and hyperprogression during immune checkpoint inhibitor therapy for urothelial and kidney cancer. World J. Urol..

[111] Seymour L., Bogaerts J., Perrone A., Ford R., Schwartz L.H., Mandrekar S., Lin N.U., Litière S., Dancey J., Chen A. (2017). iRECIST: Guidelines for response criteria for use in trials testing immunotherapeutics. Lancet Oncol..

